# Supplementary material for: Effect of Solid-State Fermentation Products of Lactobacillus plantarum, Candida utilis, and Bacillus coagulans on Growth Performance of Broilers and Prevention of Avian Colibacillosis
Source: Vet Sci. 2024 Oct 1;11(10):468. doi: 10.3390/vetsci11100468 (PMC11511520; doi:10.3390/vetsci11100468)
Supplement: Supplementary file 1 [file vetsci-11-00468-s001.zip › vetsci-3188146-supplementary.pdf]

Supplementary Materials

Figure S1: The complete fermentation process and ingredients.

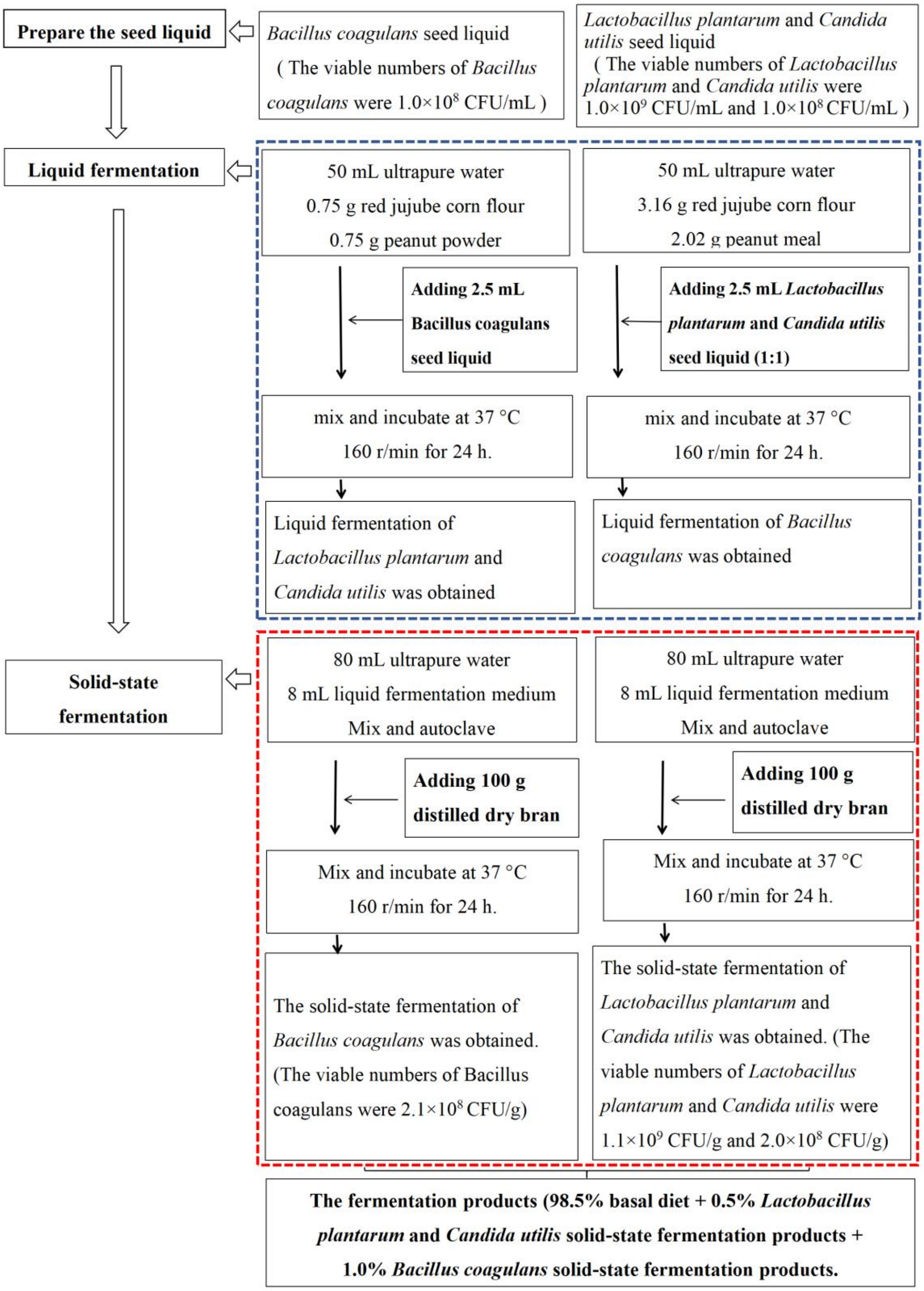

Figure S2: (a) The average daily feed intake of broilers from forty to forty-three days of age. (b) The average daily gain of broilers from forty to forty-three days of age. (c) The feed conversion ratio (FCR) of broilers from forty to forty-three days of age. Data were analyzed using ANOVA and the differences were compared using Duncan's multiple range test to the significance levels of 5% and 1%. Diverse lowercase letters show significant differences (p <0.05), diverse capital letters show significant differences (p <0.01).

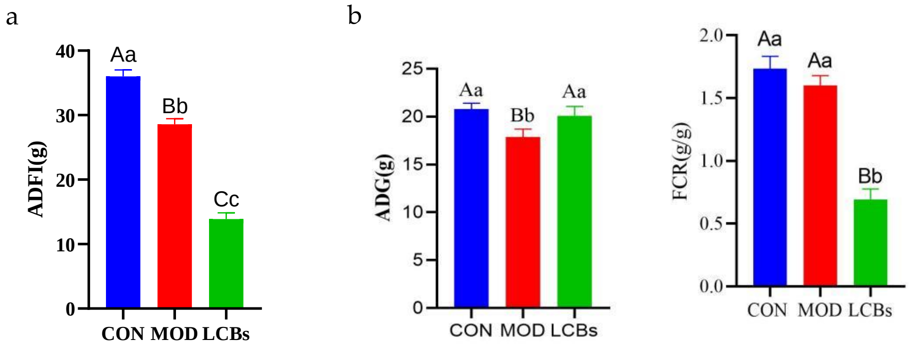

Figure S3. The composition of the intestinal flora at the level of species.

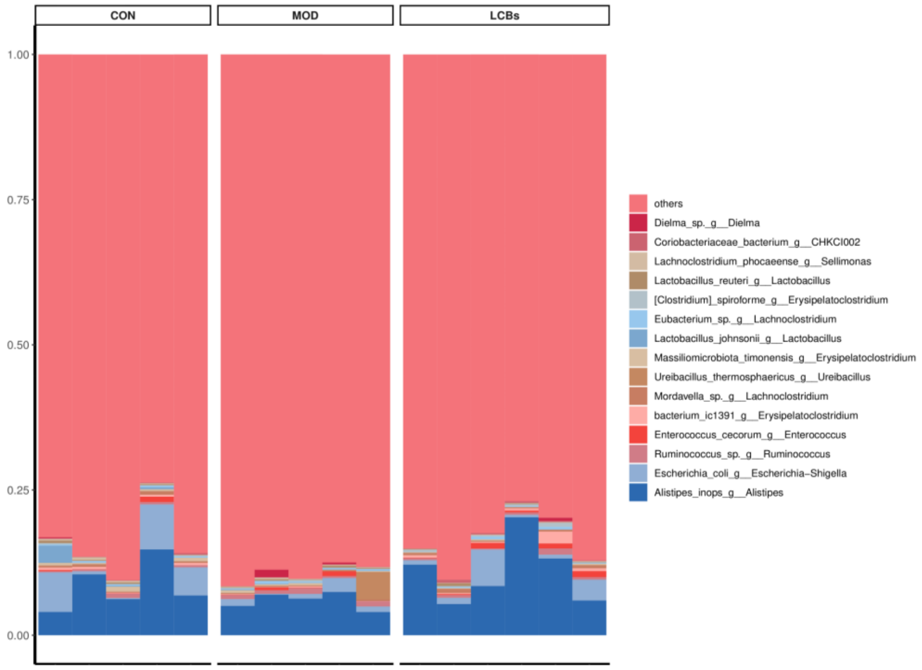

Table S1: Ingredients and nutrient composition of the basal diets

Ingredients	Percentage (%)	Nutrient composition	Percentage (%)
Corn	61.17	Metabolism energy, (MJ/kg)	12.97
Soybean meal	29.5	Crude protein (%)	20.8
Fishmeal	6.5	Available P (%)	0.45
DL-Met	0.19	Ca (%)	1.02
L-Lys•HCl	0.05	Lys (%)	1.2
Bone Meal	1.22	Met + Cys (%)	0.86
Sodium chloride	0.37		
Microelement and Vitamin Compound Premix	1		
Total	100		

Table S2: Comparison of Nutrient composition percentage of CON, MOD, and LCBs Groups

Group	Nutrient composition percentage (%)					
	Metabolism energy (MJ/kg)	Crude protein (%)	Available P (%)	Ca (%)	Lys (%)	Met + Cys (%)
CON	12.95±0.02[a]	21.3±0.21[a]	0.44±0.01[a]	1.02±0.02[a]	1.23±0.02[a]	0.87±0.01[a]
MOD	13.97±0.01[a]	20.8±0.14[a]	0.43±0.02[a]	1.02±0.03[a]	1.19±0.03[a]	0.86±0.04[a]
LCBs	13.93±0.01[a]	20.4±0.26[a]	0.45±0.01[a]	1.03±0.01[a]	1.20±0.01[a]	0.89±0.02[a]

Note: Means with different lowercase superscripts in the same column differ Significantly (p <0.05).
